# Carpal Tunnel Syndrome and Trigger Finger May Be an Early Symptom of Preclinic Type 2 Diabetes

**DOI:** 10.1097/GOX.0000000000005907

**Published:** 2024-06-14

**Authors:** Mattias Rydberg, Raquel Perez, Juan Merlo, Lars B. Dahlin

**Affiliations:** From the * Department of Hand Surgery, Lund University, Skåne University Hospital, Malmö, Sweden; † Department of Translational Medicine—Hand Surgery, Lund University, Malmö, Sweden; ‡ Unit for Social Epidemiology, Department of Clinical Sciences (Malmö), Faculty of Medicine, Lund University, Malmö, Sweden; § Center for Primary Health Research, Region Skåne, Malmö, Sweden; ¶ Department of Biomedical and Clinical Sciences, Linköping University, Linköping, Sweden.

## Abstract

**Background:**

Type 2 diabetes (T2D) is a major risk factor for carpal tunnel syndrome (CTS) and trigger finger (TF), but less is known regarding the risk of developing T2D after being diagnosed with CTS or TF. CTS and TF could be early signs of preclinical T2D, and early detection of T2D is crucial to prevent complications and morbidity. Therefore, we investigate the association between CTS/TF and T2D in an adult population without previous T2D using big data registers in Sweden.

**Methods:**

Data were collected by crosslinking five nationwide Swedish registers. Individuals aged 40–85 years on December 31, 2010, without prior overt diabetes, were included (n = 3,948,517) and followed up from baseline (ie, a diagnosis of CTS or TF) or January 1, 2011, for controls, until a diagnosis of T2D, prescription of oral antidiabetics or insulin, or end of follow-up four years after baseline. Multivariate Cox regression models were created to calculate hazard ratios for T2D.

**Results:**

In total, 37,346 (0.95%) patients were diagnosed with CTS, whereof 1329 (3.46%) developed T2D. There were 17,432 (0.44%) patients who developed TF, whereof 639 (3.67%) developed T2D. Among the controls, 2.73% developed T2D. Compared with controls, there was an increased risk of developing T2D after being diagnosed with either CTS (HR 1.35; 95% confidence interval 1.28–1.43) or TF (HR 1.21; 95% confidence interval 1.12–1.31).

**Conclusion:**

Compared with controls, a diagnosis of CTS or TF was associated with 35% and 21% higher risk for later T2D, respectively, which might indicate the existence of undetected T2D in this population.

**Takeaways**

**Question:** Type 2 diabetes (T2D) is a major risk factor for carpal tunnel syndrome (CTS) and trigger finger (TF), but less is known regarding the risk of developing T2D after being diagnosed with CTS or TF.

**Findings:** Using nationwide longitudinal data, a diagnosis of CTS or TF was associated with 35% and 21% higher risk for subsequent T2D.

**Meaning:** These findings might indicate the existence of an undetected T2D in the population with TF or CTS without a prior diagnosis of T2D.

## INTRODUCTION

Type 2 diabetes (T2D) is a rapidly increasing metabolic health disorder with life-threatening complications if not managed correctly.^1^ Recent publications have established T2D as a major risk factor for several hand surgical diagnoses, including carpal tunnel syndrome (CTS), ulnar nerve entrapment, Dupuytren disease, and trigger finger (TF) as part of the diabetic hand syndrome.^2–4^ Nevertheless, less is known regarding the risk of developing T2D after being diagnosed with common hand conditions such as CTS or TF.^5^

Because early detection of T2D is crucial to prevent both diabetes-related complications and morbidity, we hypothesized that it would be possible to investigate the risk of being diagnosed with T2D after CTS and TF in an adult population using big data registers in Sweden. Similar models for T2D have been created using the population-based data in the UK-biobank.^6^ However, to the best of our knowledge, no such model exists that uses two frequent hand surgical diagnoses. As both CTS and TF are common in a middle-aged population, a diagnosis of CTS or TF could act as a warning sign of poor metabolic control and subsequent risk of developing T2D. Furthermore, previous cross-sectional studies in an American population have shown a high prevalence of both prediabetes and T2D in individuals undergoing surgery for CTS or TF. Indeed, the prevalence of prediabetes in the group with bilateral CTS was as high as 60%.^5^

Thus, our aim here was to investigate the association between CTS, TF, and T2D using big data registers in Sweden to identify high risk individuals to facilitate early lifestyle intervention and management for these individuals. Such proactive measures could significantly reduce the burden of T2D-related complications, enhance the quality of life for the affected individuals, and contribute to the broader efforts to combat the increasing worldwide prevalence of T2D.

## POPULATION AND METHODS

### Databases

The Total Swedish Population Register and the Longitudinal Integration Database for Health Insurance and Labour Market Studies, administered by Statistics Sweden (www.scb.se/en/), as well as the National Patient Register (NPR), the Cause of Death Register and the Swedish Prescribed Drug Register, administered by the National Board of Health and Welfare (www.socialstyrelsen.se/en/), were linked together following an analogous approach as previously described.^7^ The record linkage was performed using all Swedish citizens’ unique personal identification number. The procedure was conducted in accordance with the Declaration of Helsinki and was facilitated by the National Board of Health and Welfare and Statistics Sweden after revision and consent by their own data safety committees and initial ethical approval by the Regional Committee in South Sweden (approval no.: 2014-856).

The Swedish Prescribed Drug Register contains information about all drug dispensations in the Swedish pharmacies, except from stockpiles in nursing homes and hospital wards, coded according to the Anatomical Therapeutic Chemical classification system, whereas the NPR codes discharge diagnoses from hospital and outpatient clinics according to the International Classification of Diseases and Causes of Death, 10th version (ICD-10). The NPR also records and codes clinical and surgical procedures according to the Swedish version of the Nordic Medico-Statistical Committee (https://nhwstat.org/) classification of surgical procedures. The Total Swedish Population register and the Longitudinal Integration Database for Health Insurance and Labour Market Studies database provide demographic and socioeconomic information.

### Population Sample

**Fig. 1.**
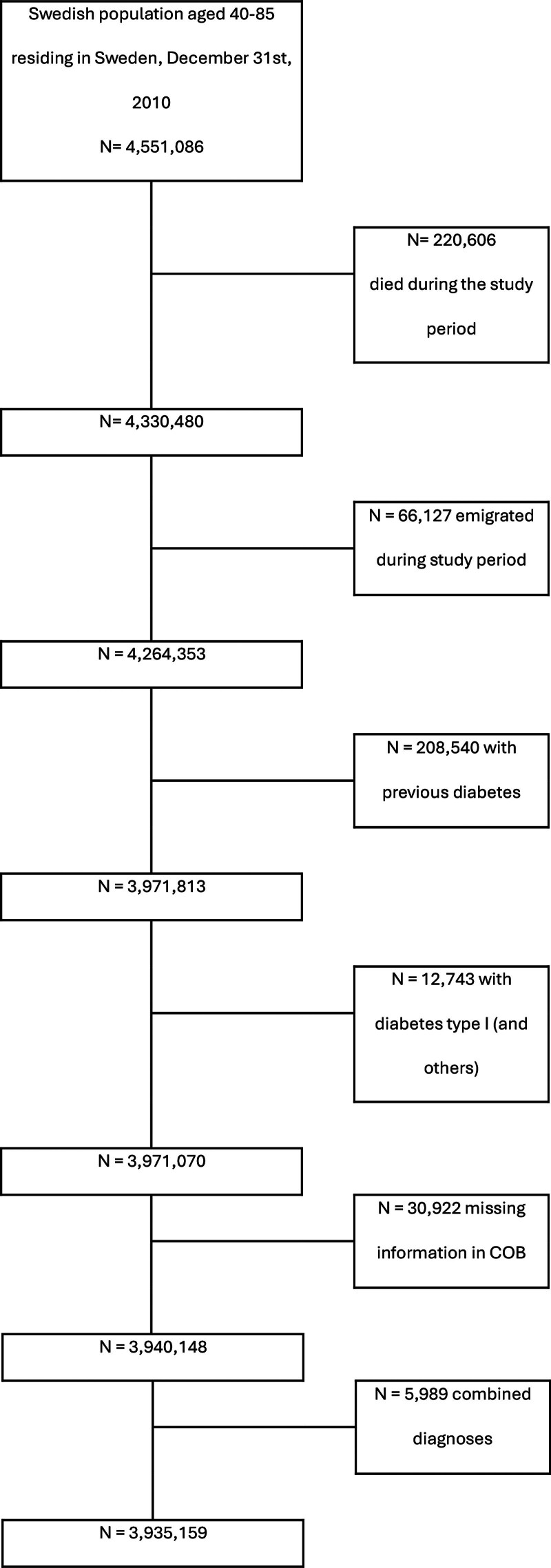
Derivation of population sample. COB, country of birth; N, no. individuals

Firstly, we identified all individuals, aged 40–85 years, residing in Sweden by December 31, 2010 (n = 4,551,086). Then, we excluded individuals who died (n = 220,606), emigrated before December 31, 2016 (n = 66,127), had previous T2D (n = 280,540), had type 1 diabetes and others (n = 12,743), had no information on country of birth (n = 30,992) and had combined CTS and TF diagnoses (n = 5989). The final population dataset consisted of 3,935,159 individuals, of whom 69,578 presented with a first diagnosis (ie, primary) for CTS or TF during the period of 2008–2012 (Fig. 1).

Every individual was assigned an individual baseline date, defined by the first diagnosis of CTS or TF between January 1, 2008, and December 31, 2012, and was followed up for 4 years from the baseline date. For individuals without a diagnosis of CTS or TF, the baseline was assigned to January 1, 2011. From baseline, individuals were followed up for 4 years until a prescription of insulin or oral antidiabetics was dispensed or a diagnosis of T2D was established. Data were also collected on previous diabetes diagnosis or prescription of insulins or oral antidiabetic drug use in the past 2 years (see below).

### Assessment of Variables

We identified individuals with a diagnosis of CTS using ICD-10 codes G560 and procedure code ACC51 (surgery), and TF using ICD-10 code M653 and procedure code NDM49. We assumed that an individual had an incident diagnosis of T2D if an ICD-10 code E11 in the NPR or at least one dispensation at the pharmacy of insulins (A10A) or oral antidiabetics (A10B) but no previous diagnosis of T2D or prescription of A10A or A10B. Age was arbitrarily classified into four wide categories: 40–49 years (reference), 50–59 years, 60–69 years, and 70–84 years. Sex was coded as man (reference) or woman according to the register. We categorized the individuals according to their country of birth into native (ie, born in Sweden; reference) or not (ie, immigrant) and obtained information on individualized disposable family income for the years 2000, 2005 and 2010 to compute a cumulative measure that considers the size of the household and the consumption weight of the individuals according to Statistics Sweden. For each of the 3 years, income levels were categorized into 25 groups (1–25) by quantiles using the complete Swedish population. These groups from the respective 3 years were summed up, so that all individuals received a value between 3 (lowest income group) and 75 (highest income group). Finally, we categorized this cumulative income by tertiles into low, middle, or high (reference) income, as previously described in several articles.^8,9^ Individuals with missing values on income during 2000 or 2005 were assigned the values for the year 2010. No individuals had missing income data for 2010.

### Statistical Analyses

Demographical and socioeconomical characteristics of the population as well as the presence of T2D or a prescription of insulins or oral antidiabetics by categories of CTS and TF were calculated (Table 1). We applied Cox regression models to investigate incident of T2D in individuals with CTS, TF, surgery for CTS, and surgery for TF, respectively, with the control population as reference. The first crude model included diagnoses of CTS or TF. The second, multivariate model added age, sex, income, country of birth, and cohabiting (Table 2). Stratified analyses by sex were also presented, as well as age-stratified analyses as supplement.

**Table 1.** Demographic and Socioeconomic Characteristics of the Population and Existence of Diabetes by the Presence or Absence of Diagnoses with or without Surgery for CTS and Trigger Finger in the Population Aged 40–85 Residing in Sweden 2010

	Diagnosis		Type			
	No, N (%)	Yes, N (%)				
			CTS	TF	CTS + Surgery	TF + Surgery
	3.865.581 (98.23)	69.578 (1.77)	37.346 (0.95)	17.432 (0.44)	11.261 (0.29)	3.539 (0.09)
Diabetes	105.345 (2.73)	2.539 (3.65)	1.329 (3.56)	639 (3.67)	425 (3.77)	146 (4.13)
Sex						
Men	1.874.838 (48.50)	23.115 (33.22)	12.145 (32.52)	6.261 (35.92)	3.490 (30.99)	1.219 (34.44)
Women	1.990.743 (51.50)	46.463 (66.78)	25.201 (67.48)	11.171 (64.08)	7.771 (69.01)	2.320 (65.56)
Age (mean SD)	57.55 (11.57)	59.17 (11.30)	57.86 (11.57)	62.16 (10.13)	58.00 (11.47)	62.04 (9.82)
40–49	1.189.213 (30.76)	15.736 (22.62)	10.488 (28.08)	1.869 (10.72)	3.306 (26.96)	343 (9.69)
50–59	1.027.802 (26.59)	21.628 (31.08)	11.533 (30.88)	5.354 (30.71)	3.618 (32.13)	1.123 (31.73)
60–69	976.877 (25.27)	18.336 (26.35)	8.522 (22.82)	5.989 (34.36)	2.544 (22.59)	1.281 (36.20)
70–84	671.689 (17.38)	13.878 (19.95)	6.803 (18.22)	4.220 (24.21)	2.063 (18.32)	792 (22.38)
Income						
Low	855.018 (22.12)	16.220 (23.31)	9.688 (25.94)	3.074 (17.63)	2.817 (25.02)	641 (18.11)
Middle	1.274.152 (32.96)	23.718 (34.09)	12.997 (34.80)	5.558 (31.88)	4.041 (35.88)	1.122 (31.70)
High	1.736.411 (44.92)	29.640 (42.60)	14.661 (39.26)	8.800 (50.48)	4.403 (39.10)	1.776 (58.18)
Country of Birth						
Immigrant	535.616 (13.86)	10.014 (14.39)	5.863 (15.70)	2.441 (14.00)	1.271 (11.29)	439 (12.40)
Native	3.329.965 (86.14)	59.564 (85.61)	34.483 (84.30)	14.997 (86.00)	9.990 (88.71)	3.100 (87.60)

**Table 2.** Cox Regression Analysis with Hazard Ratios (HR) for Incident DM in Relation to CTS. TF, CTS + Surgery and TF + Surgery

		Combined		Women		Men	
		Model 1	Model 2	Model 1	Model 2	Model 1	Model 2
Diagnoses							
	No		Reference		Reference		Reference
	CTS		1.35 (1.28–1.43)		1.41 (1.31–1.52)		1.29 (1.19–1.40)
	TF		1.21 (1.12–1.31)		1.17 (1.05–1.30)		1.27 (1.14–1.42)
	CTS + surgery		1.48 (1.34–1.63)		1.63 (1.44–1.83)		1.29 (1.10–1.51)
	TF + surgery		1.37 (1.16–1.61)		1.39 (1.12–1.74)		1..34 (1.05–171)
Sex							
	Men	1.58 (1.56–1.60)	1.58 (1.56–1.60)				
	Women	Reference	Reference				
Age							
	40–49	Reference	Reference	Reference	Reference	Reference	Reference
	50–59	2.08 (2.04–2.12)	2.07 (2.03–2.11)	2.08 (2.01–2.15)	2.07 (2.00–2.14)	2.09 (2.04–2.15)	2.09 (2.04–2.15)
	60–69	3.34 (2.28–3.41)	3.34 (3.28–3.40)	3.57 (3.46–3.68)	3.57 (3.46–3.69)	3.23 (3.15–3.31)	3.23 (3.15–3.31)
	70–85	3.56 (3.49–3.63)	3.56 (3.49–3.63)	3.94 (3.81–4.06)	3.93 (3.82–4.06)	3.28 (3.20–3.37)	3.28 (3.19–3.36)
Income							
	Low	1.42 (1.39–1.44)	1.42 (1.39–1.44)	1.64 (1.59–1.68)	1.64 (1.60–1.68)	1.28 (1.25–1.30)	1.28 (1.25–1.30)
	Middle	1.24 (1.22–1.26)	1.24 (1.22–1.26)	1.39 (1.36–1.42)	1.39 (1.36–1.42)	1.15 (1.13–1.17)	1.15 (1.13–1.17)
	High	Reference	Reference	Reference	Reference	Reference	Reference
Country of birth							
	Immigrant	1.52 (1.49–1.54)	1.52 (1.49–1.54)	1.52 (1.48–1.55)	1.52 (1.48–1.55)	1.52 (1.49–1.56)	1.52 (1.49–1.56)
	Native	Reference	Reference	Reference	Reference	Reference	Reference
ROC		0.65	0.65	0.65	0.65	0.63	0.63

The table displays HR + 95% CI adjusted for sex, age, income, and country of birth in model 2. The table also stratifies for sex.

COX regression: model 1→ logistic regression, socidemographic variables; model 2→ model 1 + type of diagnoses.

We estimated the discriminatory accuracy (DA) for each model by calculating the area under the receiver operating characteristic curve (AUC). The value of the AUC ranges from 0.5 to 1, with 1 representing perfect discrimination and 0.5 indicating no predictive accuracy. Using the criteria proposed by Hosmer and Lemeshow, we classified DA as absent or very weak (AUC = 0.5–0.6), poor (AUC >0.6–≤0.7), acceptable (AUC >0.7– ≤0.8), excellent (AUC >0.8–0.90), and outstanding (AUC > 0.90).^10^ Stata, v14.1 (StataCorp, College Station, Tex.) was used to conduct the statistical analyses.

## RESULTS

In total, 3,935,159 individuals were included in the study, whereof 107,884 individuals (2.7%) developed T2D during follow-up. There were more individuals who developed T2D in the group diagnosed with CTS (3.56%) and TF (3.67%) compared with the group without CTS/TF (2.73%). All demographic characteristics are displayed in Table 1. Adding surgery to the demographics, the percentage that developed T2D increased to 3.77% and 4.13% for CTS and TF, respectively.

**Fig. 2.**
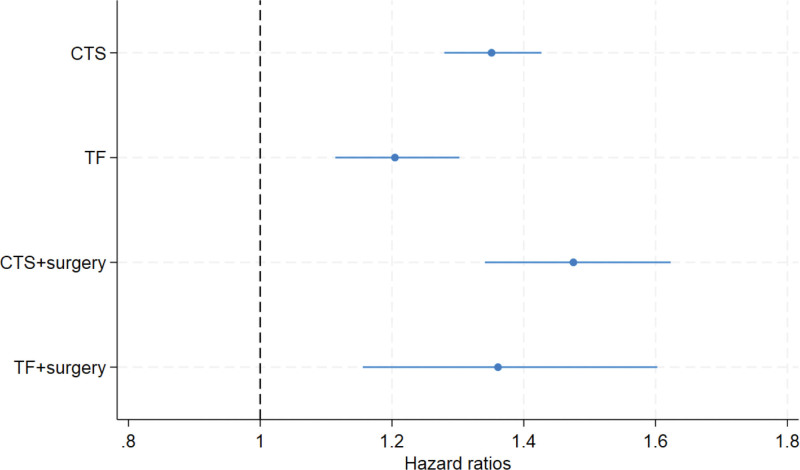
Hazard ratios (HR) with 95% CIs for respective group, adjusted for sex, age, income, and country of birth.

In the Cox regression analysis, there were an increased risk for incident T2D during follow-up for individuals who had a diagnosis of CTS [hazard ratio (HR) 1.35; 95% confidence interval (CI) 1.28–1.43] or TF (HR 1.21; 95% CI 1.12–1.31) as well as surgery for CTS (HR 1.48; 95% CI 1.34–1.63) or surgery TF (HR 1.37; 95% CI 1.16–1.61) in the fully adjusted model (Table 2, Fig. 2) compared with the control group. Furthermore, when sex-stratifying the model, there was an increased risk for developing T2D in both sex for all diagnoses (Table 2). Finally, in the age-stratified models, individuals aged 40–49 with surgery for TF had the highest risk of developing T2D during follow-up (HR 3.39; 95% CI 2.01–5.73). (**See table, Supplemental Digital Content 1**, which displays age-stratified Cox regression analysis with hazard rations and 95% CI, adjusted for sex, income levels, and country of birth. http://links.lww.com/PRSGO/D291.) The AUC values, around 0.65, are shown in Table 2 and provide information about the DA of our models.

## DISCUSSION

The major findings of this study are that individuals diagnosed with CTS or TF had, on average, a 35% and 21% higher risk of developing T2D during the 4 years of follow-up, compared with the control group. Furthermore, individuals who were diagnosed and also undergoing surgery for CTS or TF had on average a 48% and 37%, respectively, increased risk of developing T2D during follow-up. The findings were consistent for both men and women and after adjustment for age and socioeconomic variables. To our knowledge, this is the first study that uses big data registers, presenting data showing that two common hand surgical diagnoses, which are frequently observed in patients with and without T2D, may act as a warning sign for poor metabolic control and potentially can indicate high-risk individuals in a hand surgical setting. Interestingly, previous studies on patients with surgically treated CTS and later diagnosed with diabetes, irrespective of type, seem to have worse outcomes of surgery as well as more symptoms and disability (ie, higher QuickDASH) pre- and postoperatively (12 months) than those without T2D.^11^ This is in accordance with findings that peripheral neuropathy is present already at the time of diagnosis of T2D.^12^ Indeed, because early detection of diabetes or prediabetes is crucial for preventing life-threatening complications, identifying high-risk individuals in a hand surgical outpatient clinic could potentially significantly decrease the morbidity associated with T2D.

Several studies, both observational epidemiological and experimental studies including biopsies, have now concluded that T2D is a risk factor for both CTS and TF, as part of the diabetic hand syndrome, and it is now a well-established association.^3,13,14^ As for CTS, pathophysiological alterations include direct nerve damage, with a reduced number of myelinated nerve fibers making nerves more susceptible to entrapment, and nerve swelling as a result from prolonged hyperglycemia, oxidative stress and hypoxia, and thickening of the carpal ligament in T2D, limiting the functional space in the carpal tunnel and symptom development.^4,13^ In diabetes-induced TF, thickening of both the tendons and tendon sheet as well as inflammation in the tenosynovium has been proposed as biochemical explanatory alterations, resulting in the high prevalence of TF among individuals with diabetes.^3,14^

Nevertheless, as previously mentioned, far less is known about the temporality between the development of T2D after the onset of CTS or TF. Diabetes-induced pathobiological alterations in the nerve and ligaments could possibly be seen long before an established diagnosis of T2D, in analogy with peripheral neuropathy in lower limb,^12^ retinopathy and cardiovascular complications,^15^ and thus act as a warning sign for poor glycemic control and possibly also hyperlipidemia, another proposed risk factor for neuropathy.^16,17^ Because T2D can have minimal symptoms at first, there may be several years between the onset of the disease and its clinical diagnosis. As complications related to T2D can occur long before a diagnosis is established, and as both microvascular and macrovascular diseases often can be detected at the time of diagnosis,^15,18^ it is important to identify these patients as early as possible. Finally, as a high HbA1c has also been associated with increased complication rate among individuals undergoing open carpal tunnel release, diagnosing T2D at the time of surgery could possibly also lower the complication rate if treatment is rapidly administered.^19^

The findings of this study support previous research, indicating an increased risk of CTS or TF in individuals who later develop T2D, suggesting underlying metabolic dysfunction well before a clinical diagnosis of T2D.^20^ Furthermore, studies have shown a high frequency of both undiagnosed T2D and impaired glucose tolerance when screening individuals with idiopathic sensory neuropathy.^21^ Finally, screening for both thyroid dysfunction and T2D among patients undergoing surgery for CTS has been proposed as cost-effective in finding patients with undiagnosed T2D.^22^ What this study adds is data regarding TF, presenting results that TF can also act as a warning sign for metabolic dysfunction in analogy with CTS. Moving forward, studies are warranted to create effective screening programs in a hand surgical setting to transform these results from this study into actionable strategies that can benefit individuals at risk for developing T2D.

## STRENGTHS AND LIMITATIONS

There are several strengths in this study. Firstly, the study uses nationwide population data from Sweden, resulting in a large study cohort and enabling extended and precise age and sex-stratified analyses of the data. The Swedish 10-digit personal identification number is distinct for each Swedish citizen, enabling the seamless interconnection of databases and facilitating extensive epidemiological research within this geographic area.^23^ Second, the high-quality standards of the registers used to identify cases of individuals with CTS and TF, and also cases of incident T2D, contribute to the strengths of the study.^24^ The technique using prescribed antidiabetic medicines for identifying incident T2D have been used before in previous studies, and because T2D is the only indications for these medications, it has a high sensitivity.^25^ The nationwide population study design also minimizes the risk of selection bias,^26^ adding to the generalizability of the findings.^27^ Finally, the adjustment of socioeconomic status, such as income, is a strength of the study, as socioeconomic status has been shown in previous studies to affect both development of CTS and outcome after surgery for CTS.^28,29^

Nonetheless, our dataset exclusively encompasses individuals who have received diagnoses of CTS and TF from physicians in a hospital-based setting. It is worth noting that there are individuals whose hand symptoms are solely managed in primary care, only by occupational therapists, or remain untreated within the healthcare system. In light of this, the results from this study reflect clinically relevant CTS and TF, contrasting with studies that investigate prevalence within the general population, such as survey-based investigations. Additionally, as all our patients received diagnoses from physicians, our study maintains a high level of case validity when compared with studies reliant on self-reported data.

Finally, given the observational nature of the study, it is essential to note that we cannot establish causal inference or delineate specific pathophysiological mechanisms between CTS, TF and T2D, despite the results presented. Future studies should include prospective data, preferably including biopsies from individuals at high risk, possibly identifying early biochemical and histological changes in, for example, the carpal ligament,^30^ tendon sheet,^31^ posterior interosseous nerve,^32^ or surrounding connective tissue.^33^ Other potential confounding factors, such as symptom duration and severity, smoking, body mass index, and occupation, should preferably also be included in future studies, as these variables were not available in the registers used in this study.

## CONCLUSIONS

The risk of T2D was approximately 35% and 21% higher among individuals diagnosed with CTS and TF, respectively, compared with the control population during a 4-year follow-up. The results suggest that CTS and TF potentially serve as early indicators of preclinical T2D. Further studies are necessary to first validate these findings in a different population, but also include additional variables, such as body mass index, alcohol consumption, and smoking, to improve the models and to adjust for potential confounding.

## DISCLOSURES

The authors have no financial interest to declare in relation to the content of this article.

## FUNDING

This study was supported by Stiftelsen Thelma Zoégas fond för medicinsk forskning, Greta och Johan Kocks stiftelser, the Swedish Diabetes Foundation [grant no.: DIA2020-492], local funds from Skåne University Hospital and Lund University, Elly Olsson’s Foundation for Medical Research, and the Swedish Research Council [grant no.: 2021-01942].

## Supplementary Material


