# Supplementary material for: Carpal Tunnel Syndrome and Trigger Finger May Be an Early Symptom of Preclinic Type 2 Diabetes
Source: Plast Reconstr Surg Glob Open. 2024 Jun 14;12(6):e5907. doi: 10.1097/GOX.0000000000005907 (PMC11177834; doi:10.1097/GOX.0000000000005907)
Supplement: Supplementary file 1 [file gox-12-e5907-s001.pdf]

SDC 1. Age-stratified Cox regression analysis with hazard rations and 95% CI, adjusted for sex, income levels and country of birth.

		40-49	50-59	60-69	70-85
		**Combined**			
**Diagnoses**					
	No	Reference	Reference	Reference	Reference
	CTS	1.98 (1.75-2.24)	1.41 (1.27-1.56)	1.15 (1.03-1.28)	1.24 (1.12-1.38)
	TF	1.36 (0.98-1.93)	1.33 (1.14-1.55)	1.26 (1.12-1.42)	1.04 (0.89-1.20)
	CTS + surgery	1.85 (1.44-2.37)	1.49 (1.25-1.79)	1.46 (1.23-1.74)	1.36 (1.14-1.64)
	TF + surgery	3.39 (2.01-5.73)	1.15 (0.80-1.66)	1.31 (1.01-1.69)	1.35 (1.00-1.82)
